# Cell type-specific Nrf2 expression in multiple sclerosis lesions

**DOI:** 10.1007/s00401-015-1452-x

**Published:** 2015-06-19

**Authors:** Simon Licht-Mayer, Isabella Wimmer, Sarah Traffehn, Imke Metz, Wolfgang Brück, Jan Bauer, Monika Bradl, Hans Lassmann

**Affiliations:** Department of Neuroimmunology, Center for Brain Research, Medical University of Vienna, Spitalgasse 4, 1090 Vienna, Austria; Institute of Neuropathology, University of Göttingen, Göttingen, Germany

**Keywords:** Multiple sclerosis, Nrf2, Oxidative stress, Demyelination, Neurodegeneration, Fumarate, BG12

## Abstract

Oxidative injury appears to play a major role in the propagation of demyelination and neurodegeneration in multiple sclerosis (MS). It has been suggested that endogenous anti-oxidant defense mechanisms within MS lesions are insufficient to prevent spreading of damage. Thus, current therapeutic approaches (e.g., fumarate treatment) target to up-regulate the expression of a key regulator of anti-oxidative defense, the transcription factor nuclear factor (erythroid-derived 2)-like 2 (Nrf2). In this study, we show that Nrf2 is already strongly up-regulated in active MS lesions. Nuclear Nrf2 expression was particularly observed in oligodendrocytes and its functional activity is indicated by the expression of one of its downstream targets (heme oxygenase 1) in the same cells. In contrast, only a minor number of Nrf2-positive neurons were detected, even in highly inflammatory cortical lesions presenting with extensive oxidative injury. Overall, the most pronounced Nrf2 expression was found in degenerating cells, which showed signs of apoptotic or necrotic cell death. Via whole-genome microarray analyses of MS lesions, we observed a differential expression of numerous Nrf2-responsive genes, also involved in the defense against oxidative stress, predominantly in areas of initial myelin destruction within actively demyelinating white matter lesions. Furthermore, the expression patterns of Nrf2-induced genes differed between the white matter and cortical gray matter. Our study shows that in the MS brain, Nrf2 expression varies in different cell types and is associated with active demyelination in the lesions.

**Electronic supplementary material:**

The online version of this article (doi:10.1007/s00401-015-1452-x) contains supplementary material, which is available to authorized users.

## Introduction

Recent studies suggest a major role of oxidative injury in the pathogenesis of demyelination and neurodegeneration in multiple sclerosis (MS). This view is based on the presence of oxidized lipids, proteins and DNA in MS lesions [4, 43], in particular in oligodendrocytes within active lesions [21] and in neurons within areas of severe active cortical demyelination and neurodegeneration [15]. Oxidative injury is associated with the presence of activated microglia-expressing enzymes involved in oxygen radical production such as NADPH oxidases [14, 15] and myeloperoxidase [20]. Additionally, oxidative tissue damage may be aggravated via the Haber–Weiss Fenton reactions catalyzed by redox-active iron, which has been shown to be present in and around a subset of classical active and slowly expanding lesions, respectively [22]. Furthermore, mitochondrial injury, which is prominent within MS lesions and the normal-appearing cortex [8, 29], may be induced by oxidative injury but can also amplify oxidative stress [32]. The data suggest that in early MS, oxidative injury may be driven by inflammation and radical production in activated microglia and amplified in the progressive stage of MS by age- and disease burden-related additional mechanisms [27, 30].

A prominent defense mechanism counteracting oxidative stress is the induction of the transcription factor nuclear factor (erythroid-derived 2)-like 2 (Nrf2; encoded by the *NFE2L2* gene), which triggers the expression of a variety of anti-oxidative defense molecules when translocated into the nucleus [9, 10]. Experimental autoimmune encephalomyelitis was more severe in Nrf2-deficient mice in comparison with wild-type littermates [24], and induction of Nrf2 expression by treatment with fumarate was protective in this animal model [28]. For these reasons and since the induction of endogenous anti-oxidant defense mechanisms might be insufficient in MS lesions, therapeutic trials testing dimethyl fumarate for its neuroprotective effects have been initiated and in part already conducted in MS patients [16, 18, 37, 39]. So far, results show a reduction of relapses and magnetic resonance imaging (MRI) activity in patients with relapsing–remitting MS (RRMS), which was associated with a significant reduction of sustained disability progression in one of the two phase III trials.

Nrf2 has been described to be consistently up-regulated in multiple sclerosis plaques, but it was only detected in astrocytes and macrophages [41] and in some spinal cord neurons [28]. It has not been detected in oligodendrocytes so far, which are the prime target for oxidative damage within demyelinating lesions. In our present study, we report a high nuclear Nrf2 reactivity in oligodendrocytes in actively demyelinating lesions in patients with acute, relapsing as well as progressive disease. Most extensive Nrf2 expression was present in degenerating cells. Our observations raise doubts that further stimulation of Nrf2 by fumarate-induced cell stress protects oligodendrocytes in active lesions.

## Materials and methods

The study was performed on formalin-fixed paraffin-embedded (FFPE) autopsy brain tissue derived from 28 controls without neurological disease and 23 MS cases (Table 1). The MS cohort included 6 acute MS (AMS) cases defined by a clinical course leading to death within 12 months after disease onset. One patient died during disease exacerbation in the RRMS stage. Nine patients presented with secondary progressive MS (SPMS) and seven with primary progressive MS (PPMS). The control cases included patients without neurological disease and neuropathologically detectable lesions in the central nervous system. In addition we included 2 brain biopsies of progressive multifocal leukoencephalopathy (PML) patients, who were treated daily with 4 (patient 1) and 5 (patient 2) tablets of Fumaderm (120 mg dimethyl fumarate and 95 mg monomethyl fumarate) until the last day before diagnostic brain biopsy. For control reasons, we included 2 brain biopsies from PML patients without fumarate therapy (Table 1). To determine the effect of systemic inflammation on Nrf2 expression in the brain, the control cohort also included patients, who died under septic conditions. Clinical and pathological information on the patients is summarized in Table 1. This study was approved by the ethics committee of the Medical University of Vienna (EK No. 048/01/2014).

**Table 1:** Clinical demographics

Case ID	Disease course	Age	Sex	Disease duration (months)	Progressive phase duration (months)	MS-related treatment	Cause of death
MS 1	AMS	78	M	2	–	No	MSR
MS 2	AMS	34	F	4	–	S	MSR
MS 3	AMS	45	M	0.2	–	S	MSR
MS 4	AMS	69	F	2	–	S	MSR
MS 5	AMS	35	M	1.5	–	S	MSR
MS 6	AMS	45	M	1,5	–	S	MSR
MS 7	RRMS	40	F	120	–	S	UK
MS 8	SPMS	41	M	137	99	Mit, IFNβ, S	CV
MS 9	SPMS	34	M	120	n.a.	No	UK
MS 10	SPMS	42	F	241	87	No	UK
MS 11	SPMS	53	F	241	104	S	P
MS 12	SPMS	62	F	144	n.a.	S	P
MS 13	SPMS	48	F	410	182	No	UK
MS 14	SPMS	46	F	444	228	No	CV
MS 15	SPMS	56	M	372	132	No	CV
MS 16	SPMS	59	F	492	168	S	CV
MS 17	PPMS	55	F	60	60	No	PE
MS 18	PPMS	36	M	61	61	No	PE
MS 19	PPMS	71	F	264	264	No	PE
MS 20	PPMS	53	M	168	168	No	CV
MS 21	PPMS	77	F	168	168	No	CV
MS 22	PPMS	67	M	87	87	No	CV
MS 23	PPMS	54	F	72	72	No	CV
Control 1	Control	39	F	–	–	–	
Control 2	Control	36	F	–	–	–	
Control 3	Control	46	M	–	–	–	
Control 4	Control	45	F	–	–	–	
Control 5	Control	47	F	–	–	–	
Control 6	Control	29	F	–	–	–	
Control 7	Control	57	M	–	–	–	
Control 8	Control	42	F	–	–	–	
Control 9	Control	46	M	–	–	–	
Control 10	Control	72	M	–	–	–	
Control 11	Control	65	M	–	–	–	
Control 12	Control	67	M	–	–	–	
Control 13	Control	73	M	–	–	–	
Control 14	Control	71	F	–	–	–	
Control 15	Control	80	F	–	–	–	
Control 16	Control	84	F	–	–	–	
PML + fumarate 1	PML	77	M	–	–	Fumarates for psoriasis	Brain biopsy
PML + fumarate 2	PML	68	M	–	–	Fumarates for psoriasis	Brain biopsy
PML control 1	PML	60	M	–	–	–	Brain biopsy
PML control 2	PML	85	M	–	–	–	Brain biopsy
Septic control 1	Septic control	97	F	–	–	–	
Septic control 2	Septic control	37	M	–	–	–	
Septic control 3	Septic control	71	F	–	–	–	
Septic control 4	Septic control	45	M	–	–	–	
Septic control 5	Septic control	74	M	–	–	–	
Septic control 6	Septic control	51	F	–	–	–	
Septic control 7	Septic control	70	M	–	–	–	
Septic control 8	Septic control	71	M	–	–	–	
Septic control 9	Septic control	95	F	–	–	–	
Septic control 10	Septic control	42	F	–	–	–	
Septic control 11	Septic control	89	F	–	–	–	
Septic control 12	Septic control	79	F	–	–	–	

*MS* multiple sclerosis, *AMS* acute multiple sclerosis, *RRMS* relapsing–remitting multiple sclerosis, *SPMS* secondary progressive multiple sclerosis, *PPMS* primary progressive multiple sclerosis, *F* female, *M* male, *n.a.* not available

MS-related treatment (last month before death): *No* no immunosuppressive or immunomodulatory therapy; *S* steroids, *Mitox* mitoxantrone; *IFN-β* β-interferon

Cause of death: *MSR* directly MS related, *CV* cardiovascular, *PE* pulmonary embolism, *P* pneumonia, *UK* unknown

### Neuropathology and immunohistochemistry

All disease and control autopsy cases underwent detailed neuropathological examination of multiple tissue blocks covering various brain regions. Lesion activity was evaluated as previously described [17, 26]. Routine immunohistochemistry was performed on paraffin sections according to established techniques [2, 25]. For a detailed list of primary antibodies, dilutions and respective pre-treatment of tissue sections see Table 2. For Nrf2 stainings, we used two different primary antibodies, which resulted in very similar staining patterns. However, in combination with FFPE tissue, the antibody from Abcam showed a bit higher degree of sensitivity and less background staining and was, thus, used for quantitative analysis of Nrf2 expression. Cells with nuclear DNA fragmentation were identified via TUNEL staining [19].

**Table 2:** Primary antibodies and antigen retrieval

Primary antibody	Antibody type	Target	Source	Staining
CA II	Sheep (pAB)	Carbonic anhydrase II	PC076; BindingSite	1:1500; E
CD68	Mouse (mAB)	Cluster of differentiation 68	M0814; Dako	1:100; E
E06	Mouse (mAB)	Oxidized phospholipids	[34]	10 µg/ml; none
GFAP	Mouse (mAB)	Glial fibrillary acid protein	0410080; ThermoSc	1:200; E
HLA-DP, DQ, DR	Mouse (mAB)	Human leukocyte antigen	M0775; Dako	1:100; E
HO-1	Rabbit (pAB)	Heme oxygenase-1	ab13243; Abcam	1:2000; CSA; C
KEAP1	Mouse (mAb)	Kelch-like ECH-associated protein 1	NBP2-03319, Novus Biologicals	1:250; E
MAG	Mouse (mAB)	Myelin-associated glycoprotein	ab89780; Abcam	1:1000; E
MAP 2	Mouse (mAB)	Microtubule-associated protein 2	M4403; Sigma	1:100; E
Nrf2	Rabbit (mAB)	Nuclear factor (erythroid-derived 2)-like 2	ab62352; Abcam	1:100; E
Nrf2	Rabbit (pAB)	Nuclear factor (erythroid-derived 2)-like 2	C-20; Santa Cruz	1:500; C
p22phox	Rabbit (pAB)	NADPH oxidase	Sc-20781; Santa Cruz	1:100; C
PLP	Mouse (mAB)	Proteolipid protein	MCA8394; AbD Serotec	1:1000; E
Tppp/p25	Rat (pAB)	Tubulin polymerization-promoting protein	[23]	1:100; E

*mAB* monoclonal antibody, *pAB* polyclonal antibody, *C* citrate buffer pH 6, *E* ethylenediaminetetraacetic acid buffer pH 9.0

For fluorescent double and triple labeling of Nrf2 with cell type-specific markers and heme oxygenase 1, tissue sections were routinely dewaxed and rehydrated. After the respective antigen retrieval, primary antibodies for Nrf2 and cell type-specific markers were applied simultaneously at 4 °C overnight. After washing with TBS, secondary antibody mixes consisting of biotinylated-anti-rabbit (Jackson ImmunoResearch, 1:2000) and anti-mouse, -rat, -sheep or -goat antibodies conjugated to Cy3 or Cy5 (Jackson ImmunoResearch; 1:100) were applied simultaneously for 1 h at room temperature. Subsequently, the staining procedure was finished by incubation with streptavidin-Cy2 (Jackson ImmunoResearch; 1:100) for 1 h at room temperature. Fluorescent preparations were examined using a Leica SP2 confocal laser scan microscope (Mannheim, Germany).

### Quantitative analysis of Nrf2 expression and statistical evaluation

For quantitative analysis, all respective tissue slides were stained for Nrf2 at the same time. This allowed not only to separate cells with intense versus weak expression within different areas of a single section, but also between sections of different MS or control cases. The following regions of interest were analyzed in the white matter: In and around active white matter plaques defined by the presence of macrophages with early myelin degradation products [5], we differentiated between the normal-appearing white matter (NAWM; at least 1 cm distant from the plaque edge), the periplaque white matter (PPWM; 0.2–0.5 mm distant from the plaque edge), areas of initial demyelination (prephagocytic area characterized by profound microglia activation, scattered macrophages with early myelin degradation products, myelin sheaths with initial stages of dissolution and oligodendrocyte apoptosis) [1, 26] and the demyelinated plaque center densely packed with macrophages containing early and late myelin degradation products [5]. In slowly expanding (smoldering) plaques, NAWM and PPWM were defined similarly as in active plaques. The zone of active demyelination was defined by the presence of numerous activated microglia and scattered macrophages with early myelin degradation products. Additionally, the inactive plaque center was analyzed as well. Inactive lesions were defined by their sharp plaque edge, the absence of a rim containing activated microglia and the lack of macrophages with myelin degradation products. In addition, we quantified neurons with nuclear Nrf2 expression in the cerebral cortex of MS patients with progressive disease and presence of cortical lesions, control cases and in biopsies from patients with progressive multifocal leukoencephalopathy with or without treatment with fumarates. Nrf2-positive nuclei were counted at the microscope using the 40× objective with a counting grid in one of the ocular lenses. In the white matter, 10 visual fields of 0.25 mm^2^ were counted, thus the values shown in the figures represent cells per 2.5 mm^2^. Separate counts are provided for different regions of interest in white matter lesions. Analysis of Nrf2-positive neurons was performed in the same way as described before for white matter lesions. However, since we did not see differences in the presence of Nrf2-positive neurons within and outside cortical lesions in MS, the values were pooled per case.

Statistical analysis was performed with nonparametric tests. Statistical difference between two groups was assessed by Mann–Whitney *U* test. For the comparison of multiple groups with controls, the Kruskal–Wallis test was used, followed by pairwise Mann–Whitney *U* tests and Bonferroni–Holm correction. A *P* value smaller or equal to 0.05 was considered as statistically significant. Data were presented as scatter plots showing all actual data points as well as the median of each group.

### Whole-genome microarrays

For gene expression analysis of Nrf2-responsive genes in active white matter and cortical lesions, we used whole-genome microarray datasets already created in previous studies [14, 15, 22]. For microarray analysis of active white matter lesions, four cases with acute MS (MS2, MS3, MS4, MS6) and four normal controls were chosen [14, 22]. From the MS material, three different regions of interest were microdissected: (a) NAWM (at least 0.5 mm apart from the lesion edge); (b) zone of initial demyelination showing partial demyelination and tissue infiltration with macrophages containing early myelin degradation products; and (c) demyelinated plaque centers, which were still densely packed with macrophages containing late myelin degradation products (late active lesions; [5]). For microarray analysis of cortical lesions, three patients with secondary progressive MS (MS8, MS10, MS13), who showed active demyelination in the cerebral cortex, and three controls without neurological disease and brain lesions were chosen [15]. At least the outer four cortical layers of lesions with subpial demyelination or all cortical layers were microdissected from MS or control tissue, respectively. The cortical lesion sample contained material from one MS patient (MS10, previously described as MS1 in [15]), who presented with epileptic seizures prior to death and showed highly inflammatory active subpial lesions in many cortical regions. Data from this patient were additionally analyzed separately since they derived from lesions with an extreme degree of active inflammation, microglia activation and oxidative injury. RNA isolation, amplification and microarray technology have been described in detail in previous studies [14, 15]. All microarray datasets have been deposited at NCBI’s Gene Expression Omnibus (GEO accession numbers GSE32915 and GSE32645).

## Results

### Nuclear Nrf2 expression is increased around active demyelinating MS lesions

**Fig. 1:**
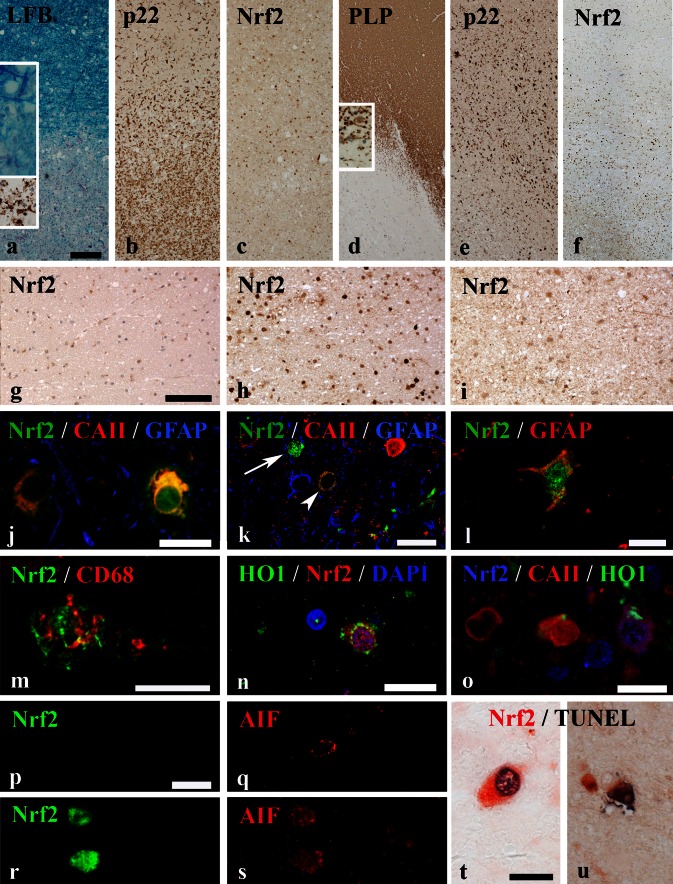
Nrf2 expression in multiple sclerosis white matter lesions. **a**–**c** Active demyelinating lesion of an acute MS case (case MS 5). **a** A decreased Luxol fast blue-periodic acid Schiff myelin staining indicates sites of ongoing demyelination. The *insets* show myelin degradation products in the Luxol fast blue staining (*blue*) and the PLP staining (*brown*). **b** In the same area, abundant activated microglia are stained for p22phox (NADPH oxidases). **c** The Nrf2 staining displays an increased nuclear expression in oligodendrocyte-like cells around the active lesion edge decreasing towards both the NAWM and the lesion center. Smoldering active demyelinating lesion of a PPMS case (MS 21). **d** Sites of demyelination are detectable by a loss of PLP staining. The *inset* shows myelin degradation products. **e** The p22phox staining illustrates abundant activated microglia around the lesion. **f** At the active lesion edge, an increase in nuclear Nrf2 accumulation can be observed, while Nrf2 expression decreases towards the NAWM and lesion center. Higher magnifications of NAWM (**g**), active lesion edge (**h**) and lesion center (**i**) highlight the differences in Nrf2 expression between the different zones in more detail (case MS 22). Within the active lesion edge (**h**) abundant cells with oligodendrocyte morphology with high nuclear accumulation of Nrf2 are present. **j** Fluorescent triple labeling of Nrf2 (*green*), CA II (*red*) and GFAP (*blue*) shows oligodendrocytes with cytoplasmic (*left*) and nuclear accumulation (*right*) of Nrf2 around the active lesion edge. **k** The same fluorescent labeling as in (**j**) illustrates a cell with extremely high nuclear Nrf2 accumulation, which has lost cellular marker protein expression (*arrow*). Additionally, a cell with cytoplasmic Nrf2 accumulation and only moderate expression of CAII (*arrowhead*) is depicted. The red-stained cell on the right represents a normal oligodendrocyte with strong CAII reactivity and only minor granular Nrf2 reactivity. **l** Fluorescent double labeling of Nrf2 (*green*) and GFAP (*red*) shows an astrocyte with cytoplasmic and nuclear Nrf2 accumulation. **m** Fluorescent double labeling of Nrf2 (*green*) and CD68 (*red*) shows cytoplasmic and nuclear Nrf2 reactivity in a macrophage. **n** Double staining of Nrf2 (*red*) and HO1 (*green*) reveals expression of both antigens in the same cell. **o** Triple staining of Nrf2 (*blue*), CaII (*red*) and HO-1 (*green*) shows coexpression of all markers in the *right-hand cell*, HO-1 expression in another CAII-positive oligodendrocyte (*middle cell*) and a single CAII-positive oligodendrocyte (*left cell*). **p**–**s** AIF staining (*red*) in relation to Nrf2 expression (*green*). **p** and **q** depict serial images of a cell, which is negative for Nrf2 but presents with a punctate mitochondrial staining for AIF. **r** and **s** show two cells with massive cytoplasmic and nuclear Nrf2 staining (**r**). In these cells, AIF is in part released from mitochondria into the cytoplasm and has translocated into the nucleus (**s**). **t**, **u** Double staining for Nrf2 and TUNEL depict cells with strong cytoplasmic and nuclear Nrf2 expression (*red*), which show nuclear DNA fragmentation (*black*) in an early (**t**; case MS 21) and late stage (**u**) of cell death (case MS 1). *Scale bars* **a**–**f** = 300 µm; **g**–**i** = 100 µm; **j**–**u** = 20 µm

**Fig. 2:**
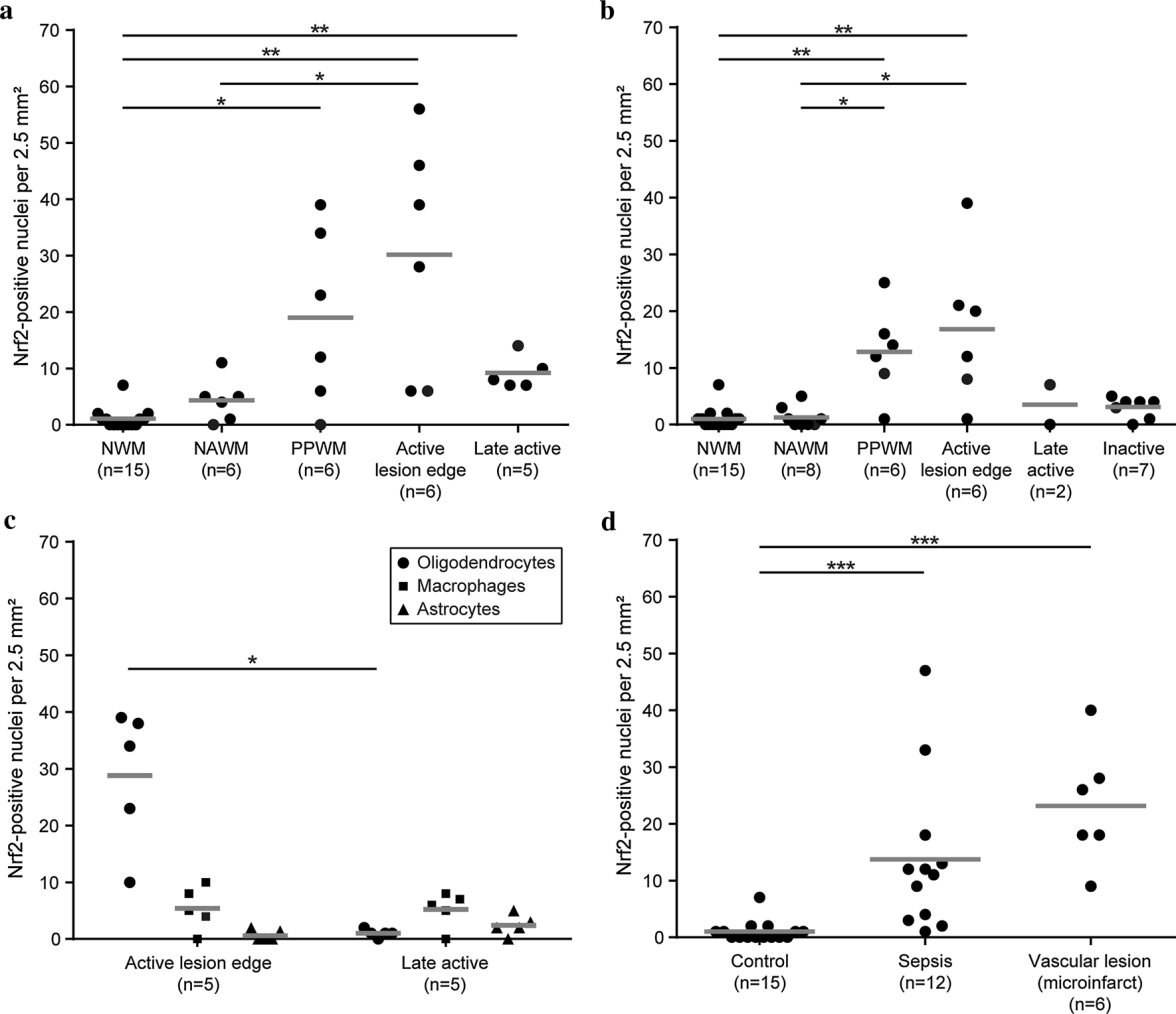
Nrf2 expression in MS lesions and controls. **a**, **b** Nrf2 expression in the normal white matter of controls (NWM), in the normal-appearing white matter of MS patients (NAWM), in the periplaque white matter (PPWM), at the active lesion edge and in the late active or inactive lesion center. Nrf2 expression is significantly increased in the PPWM and at the active edge of active demyelinating lesions in patients with acute MS and RRMS (**a**) and in slowly expanding lesions in patients with PPMS and SPMS (**b**), in comparison with normal white matter of controls. **c** The average numbers of Nrf2-positive oligodendrocytes, astrocytes and macrophages at the edge of active lesions and in the demyelinated (late active) lesion center are depicted. **d** In comparison with the white matter of unaffected control patients, Nrf2 expression is increased in control patients with concomitant microinfarcts and in control patients without pathological alteration in the brain or spinal cord, who died under septic conditions. *Scatter plots* depict all actual data points (*black dots*) as well as the median of each group (*gray bars*) *P < 0.05; **P < 0.01; ***P < 0.001

In the normal white matter (NWM) of controls and the NAWM of MS patients, only few cells were positive for Nrf2 (Fig. 1a–g). Nrf2 reactivity was found either in the cytoplasm or in addition in the cell nucleus. For quantification, only cells with a nuclear Nrf2 reactivity were counted. In the PPWM, a clear nuclear accumulation of Nrf2 was present gradiently increasing towards the zone of initial demyelination (prephagocytic lesion area [1]) at the active lesion edge (Fig. 1c, f, h). Nrf2 immunoreactivity was mainly detected in small cells with oligodendrocyte morphology and in lower incidence in cells with astrocyte or macrophage phenotype. In the demyelinated part of the lesions [5], in which myelin was completely destroyed and myelin fragments were present within phagocytes (defined as late active lesion area; Fig. 2), the staining for Nrf2 was low and comparable to the extent seen in the NAWM (Fig. 1a–f, i). This expression pattern was also reflected in quantitative analyses of Nrf2-immunoreactive cells (Fig. 2a, b). The Nrf2 expression patterns were similar in classical active lesions in acute MS and RRMS (Fig. 2a) and smoldering lesions in SPMS and PPMS (Fig. 2b). However, the absolute numbers of cells with a nuclear Nrf2 accumulation was higher in acute MS and RRMS cases compared with PPMS/SPMS cases.

Within the cytoplasm, Nrf2 is bound to Kelch-like ECH-associated protein 1 (KEAP1), which facilitates its degradation and Nrf2 becomes active after dissociation [10]. We found KEAP1 reactivity within neurons and different glia cells within the white and gray matter of MS patients and control cases. KEAP1 immunoreactivity was increased at the edge of active lesions, but this reactivity was restricted to the cytoplasm and in contrast to Nrf2 not seen in the nucleus (Suppl. Fig. 1), where active Nrf2 acts as a transcription factor.

### Oligodendrocytes are the dominant cells showing nuclear Nrf2 reactivity in active MS lesions

To determine which cell types expressed Nrf2 in MS lesions, we performed immunohistochemical double and triple stainings using confocal laser microscopy. Nrf2 expression was detected within MS lesions in oligodendrocytes (Figs. 1j, k, 2c), astrocytes (Fig. 1l), macrophages (Fig. 1m) and neurons (Fig. 4c, d). There were, however, quantitative differences in the numbers of positive oligodendrocytes, astrocytes and macrophages between different lesion stages. At the edge of active demyelinating lesions, the vast majority of Nrf2-positive cells showed morphological features of oligodendrocytes (Fig. 2c) and most of these cells were double stained with CAII (Fig. 1j). A lower number of cells co-stained with GFAP (astrocytes; Fig. 1l) or CD68 [macrophages/microglia (Fig. 1m)]. The latter cells were mainly seen in the demyelinated portion of late active lesions, which showed oligodendrocyte loss and the presence of macrophages containing myelin degradation products (Fig. 2c).

### Most intense Nrf2 reactivity is found in degenerating cells with nuclear translocation of apoptosis-inducing factor (AIF)

At the active lesion edge, an additional cell population with a very intense Nrf2 expression, which no longer allowed the differentiation between nucleus and cytoplasm, was present (Fig. 1k). These cells represented in average 4.6 % of cells with nuclear Nrf2 reactivity in active lesions of acute MS and 2.5 % in expanding lesions in progressive MS. Cell size and shape were suggestive of oligodendrocytes, however, these cells did neither express any markers for oligodendrocytes nor for astrocytes, microglia or neurons (Fig. 1h, k). Loss of cellular marker protein expression is typically seen in conditions of acute apoptotic or necrotic cell death. Cell death in oligodendrocytes in MS lesions is associated with the liberation of apoptosis-inducing factor (AIF) from mitochondria and its translocation into the nucleus [44] as well as with nuclear DNA fragmentation in the absence of caspase 3 activation. Via fluorescent double labeling, we detected a cytoplasmic and nuclear co-localization of Nrf2 and AIF in these highly Nrf2-positive cells (Fig. 1p–s). Furthermore, fragmented nuclear DNA was occasionally seen in highly Nrf2-positive cells by TUNEL staining (Fig. 1t, u).

### Functional activity of Nrf2 is suggested due to a differential expression of target genes

**Fig. 3:**
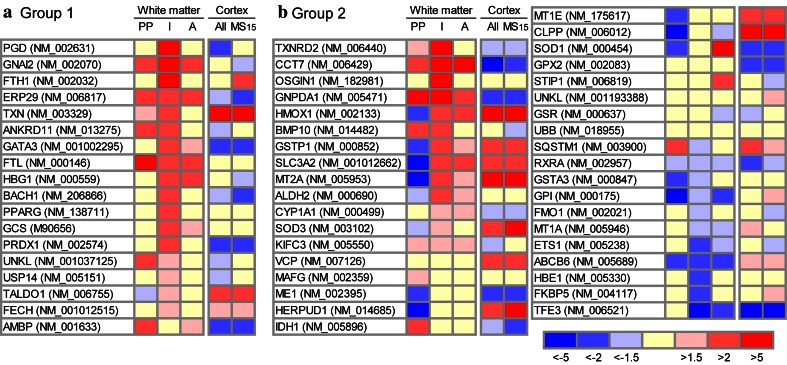
Expression of Nrf2-responsive genes in white matter and cortical MS lesions. The heat maps show color-coded fold changes of Nrf2-responsive genes in the periplaque white matter (“PP”), initial (prephagocytic) lesions (“I”) and active demyelinating lesions (“A”) as well as in active cortical lesions of all evaluated MS cases (“All”) or of the single case MS15 (“MS15”). Transcriptional changes were calculated by comparing the mean expression values of the different white and gray matter areas with the mean expression values of normal white matter or normal gray matter from control cases, respectively. **a** Group 1 represents Nrf2-responsive genes that show a low basal expression in the NWM of controls, but were up-regulated in the zones of initial demyelination, while their expression in general was lower in the NAWM and the demyelinated part of the active lesion. **b** Group 2 consists of genes, which are expressed in high levels in the NWM of controls. Half of the genes within the second groups show an increased expression in the area of initial demyelination, while a lower fraction was down-regulated in the same lesion area. In contrast, no lesion activity-associated pattern of gene expression was seen in the cortex. *PP* periplaque white matter, *I* initial (prephagocytic) lesion, *A* active demyelinating lesion

To determine the activity of Nrf2, we examined the expression of Nrf2-responsive genes (respective references for each gene are given in Supplementary Table 1). In the white matter, the most pronounced changes in gene expression were seen in microdissected areas of initial lesions. Thus, up-regulation of Nrf2 responsive genes was seen in exactly the same lesion area, which also showed the most profound expression and nuclear translocation of Nrf2 by immunohistochemistry (Figs. 2, 3). As summarized in Supplementary Table 1, we found three different response patterns, two of them further depicted in Fig. 3. The first group of genes (Group 1) showed a low basal expression in the NWM of controls, but was up-regulated in active white matter lesions in particular in the zones of initial demyelination. The second pattern (Group 2) included genes, which were already expressed in high levels in the NWM of controls. In this group, about half of the genes showed an increased expression in the area of initial demyelination, while a lower fraction was unchanged or down-regulated. Finally, a number of genes (Supplementary Table 1; Group 3) showed a very low basal expression in the NWM of controls and did not reveal any dynamic expression changes in MS lesions. They were either not expressed within the central nervous system at all, or were for technical reasons false negative in the microarrays derived from paraffin material as already extensively discussed before [14, 15].

**Fig. 4:**
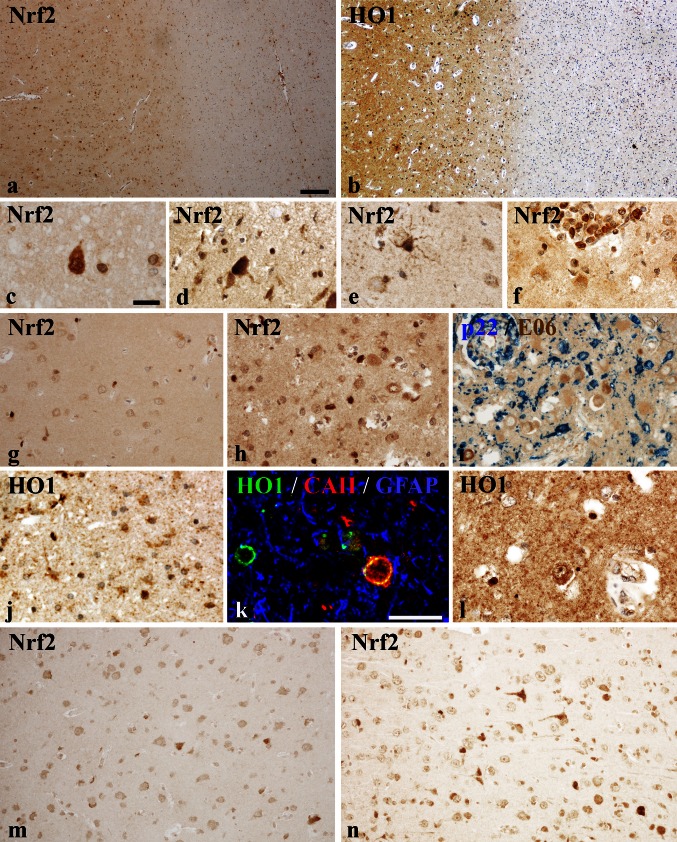
Nrf2 and HO1 expression in the cortex of MS patients and controls determined by qualitative immunohistochemistry. Low-magnification figure showing Nrf2 (**a**) and HO1 (**b**) expression in the cortex (*left-hand side*) and adjacent white matter (*right-hand side*). In cortical areas derived from both, controls (**a**; case CO 1) and MS patients (**b**; case MS 22), a higher cellular and diffuse expression of these antigens in comparison with the adjacent white matter is apparent. Examples of cellular expression of Nrf2 in the cortex; **c** neuronal cytoplasmic and nuclear expression in MS cortex; (**d**; case MS 1): neuron with strong cytoplasmic and nuclear Nrf2 reactivity in the cortex of an MS patient (case CO 8); **e** Nrf2 expression in a reactive astrocyte in a cortical lesions of an MS patient (case MS 8); **f** Nrf2 expression in inflammatory macrophages in a perivascular cuff in an active cortical MS lesion (case MS 10); **g** Nrf2 expression in small oligodendrocyte-like cells in the cortex of a control patient (case MS 20). **h**, **i** Nrf2 expression in a highly active cortical MS lesions is predominantly seen in oligodendrocytes (**h**), despite extensive NADPH oxidase (p22phox subunit) expression in microglia (*blue*) and extensive accumulation of oxidized phospholipids (E06; *brown*) in cortical neurons (case MS 10). HO-1 expression in the MS cortex is variable between cases and regions and can be seen in astrocytes (**j**; case MS 22), in oligodendrocytes (**k**, MS 22) and in neurons (**l**; MS 22). **m**, **n** Nrf2 expression in cortical neurons in two patients with PML under fumarate treatment showing either single positive neurons (**m**; case PML Fumarate 1) or small clusters of immunoreactive neurons, mainly consistent of shrunken cells with condensed nuclei (case PML Fumarate 2). *Scale bars* **a**, **b** = 100 μm; **c**–**j** and **l**–**n** = 30 μm; **k** = 20 μm

Heme oxygenase (HO-1; encoded by the *HMOX* gene), a downstream target of Nrf2 [11], was one of the genes, which was up-regulated in initial white matter lesions (Fig. 3b). It cleaves heme into CO, ferrous iron and biliverdin, which is subsequently converted to bilirubin, a potent anti-oxidant. Via immunohistochemistry, we found an expression profile of the HO-1 protein similar to that of Nrf2. Most of the cells expressing HO-1 morphologically resembled oligodendrocytes and expressed the oligodendrocyte marker CAII (Figs. 1n, o, 4k). Quantitative analysis of fluorescent double labeling of HO-1 and Nrf2 showed a co-localization of Nrf2 and HO-1 within 68 % of the immunostained cells, whereas 17 and 15 % were single-positive for HO-1 and Nrf2, respectively. Similar to Nrf2, some HO-1-positive cells were identified as macrophages or astrocytes as well (Fig. 4j).

### Neuronal expression of Nrf2 and HO-1 is minor even in highly active gray matter lesions

**Fig. 5:**
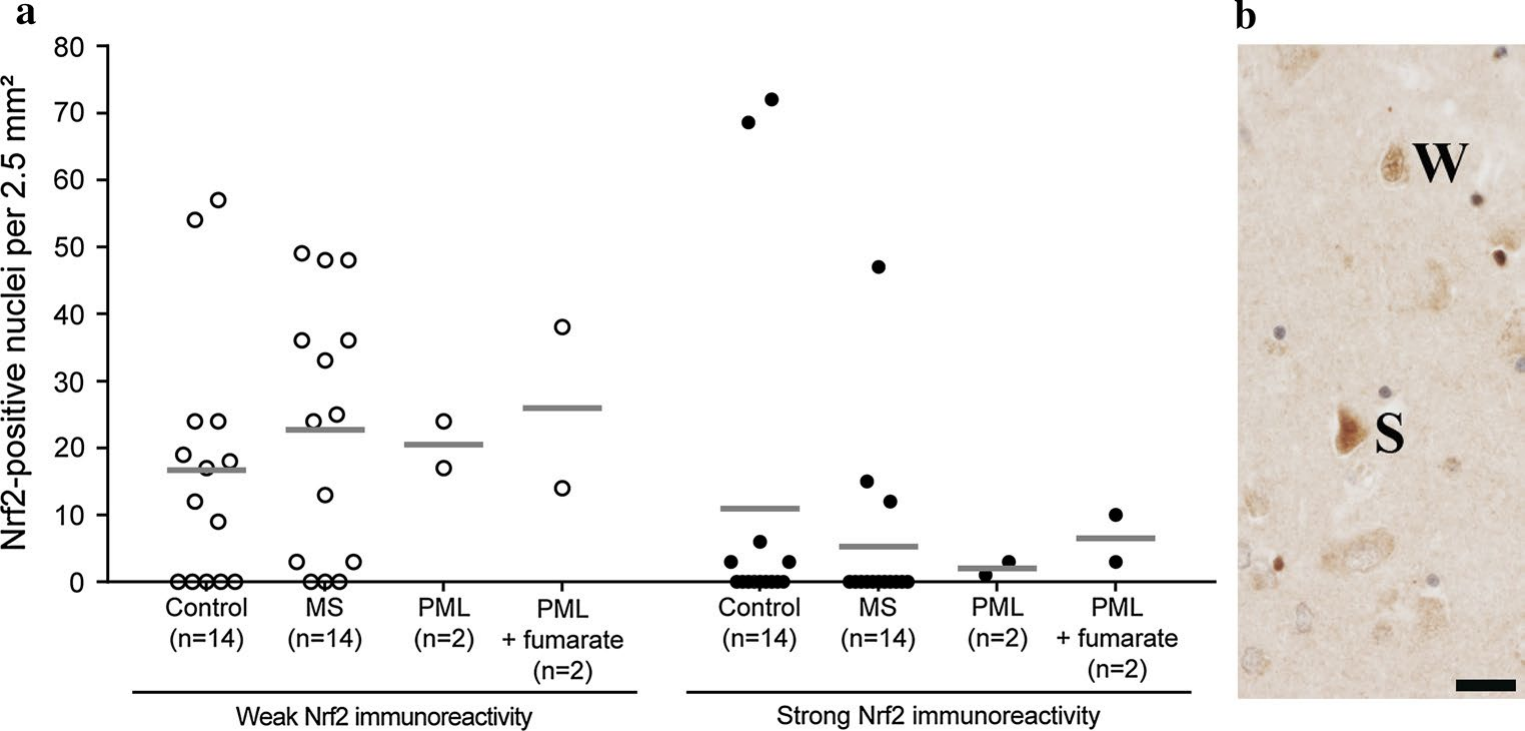
Nrf2 expression in cortical neurons. Based on the level of Nrf2 immunoreactivity (cytoplasmic and nuclear), we distinguished between two types of Nrf2-positive neurons: neurons with weak and neurons with strong Nrf2 expression. Examples are shown in (**b**) and labeled as weak (W) or strong (S). **a** Grouped according to Nrf2 immunoreactivity (weak or strong), a quantitative comparison of Nrf2-positive cortical neurons revealed no significant differences between control, MS, PML and fumarate-treated PML cases within each group. In addition to the actual data points (*open and closed circles*), the median of each data set is indicated by a *gray bar*. *Scale bar* 50 µm

Global Nrf2 expression was higher in the cortex compared to white matter of control and MS cases (Fig. 4a), however, this higher expression was mainly due to a diffuse cytoplasmic immunoreactivity in the neuropil. Even in controls, Nrf2 was already detectable in the cytoplasm of glial cells and neurons (Fig. 4c–f) and the expression levels were highly variable between cases. A similar variability of Nrf2 expression in the normal-appearing cortex was also seen in MS patients and we did not find increased neuronal reactivity between MS and control cases (Fig. 5). In some MS and control patients, a subset of neurons showed an intense immunoreactivity and Nrf2 accumulation in the nucleus (Figs. 4c, d, 5). Such neurons showed condensed and hyperchromatic nuclei and eosinophilic cytoplasm, reminiscent of acute hypoxic damage. In active demyelinating cortical lesions, strong Nrf2 immunoreactivity was present in oligodendrocytes and occasionally in astrocytes (Fig. 4h). Nrf2 reactivity within neurons was similarly low as in the cortex of normal controls and the normal-appearing MS cortex, even in highly inflammatory cortical lesions with extensive NADPH oxidase expression in microglia, massive accumulation of oxidized lipids in the neuronal cytoplasm and signs of neuronal degeneration, despite high expression of Nrf2 in adjacent cells with oligodendrocyte morphology (Fig. 4h, i). A similar pattern of expression was seen in sections stained for HO-1. In contrast to the white matter, a weak diffuse reactivity for HO-1 was present in the cytoplasm of neurons and astrocytes (Fig. 4b, j, k) in the control and MS cortex. Strong HO-1 reactivity was mainly seen in oligodendrocytes (Fig. 4k). Dystrophic or transected axons with disturbed axonal transport did not contain HO-1, despite the massive accumulation of other neuronal proteins such as amyloid precursor protein. These data suggest that in contrast to oligodendrocytes, Nrf2 induction in neurons in response to oxidative injury in MS lesions was sparse.

These data are in line with the results obtained by gene expression analysis using whole-genome microarrays (Fig. 3). Only few Nrf2-responsive genes were up-regulated in active cortical lesions in comparison to control cortex, while the majority were unchanged or down-regulated. Furthermore, a separate analysis of case MS10 presenting with extensive inflammation, active demyelination and oxidative injury did not reveal more prominent up-regulation of the respective genes in comparison with the other cortical MS cases presenting with low demyelinating activity and a lower extent of oxidative injury.

### Fumarate treatment of PML patients was not associated with increased Nrf2 reactivity in neurons

The effect of fumarate treatment in lesions was investigated in biopsies of a patient with multiple sclerosis and in 3 cases with PML, as previously described in Metz et al. [31]. In comparison to controls, this study showed a sixfold increase of cells with nuclear Nrf2 reactivity (mainly astrocytes) in the inactive MS lesion as well as in the PML lesions, but Nrf2 expression in the cortex has not been analyzed. We thus focused in the present study on neuronal Nrf2 expression in the normal appearing and not PML-affected cerebral cortex included in the biopsy tissue of the same PML cases. Fumarate treatment did not lead to an up-regulation of Nrf2 in cortical neurons (Fig. 5). Instead, the number of Nrf2-positive neurons was in the range of untreated PML patients, MS patients and control subjects. Similar to control and MS cases, a variable proportion of neurons contained cytoplasmic and some nuclear reactivity. Strong nuclear reactivity was seen mainly in neurons with alterations of acute hypoxic injury such as condensed and hyperchromatic nuclei and eosinophilic cytoplasm (Figs. 4d, 5).

### Nrf2 expression in the white matter is increased by concomitant presence of brain hypoxia or systemic inflammation

In white matter microinfarcts, which are occasionally present within the CNS of control patients without neurological disease, we observed an increased Nrf2 expression in oligodendrocytes and sparse astrocytes (Fig. 2d). Thus, we included only MS cases without any concomitant neuropathological alterations in our MS samples. Further analyses showed that in control patients, who died under septic conditions but did not reveal any inflammatory alterations in the brain or spinal cord, increased numbers of Nrf2-positive cells were found within the NWM (Fig. 2d). For this reason, MS patients (*n* = 4) who died in the course of systemic inflammatory diseases (e.g., pneumonia) were separately analyzed regarding Nrf2 expression in the NAWM and lesions. These patients showed the same lesion-associated patterns of Nrf2 expression compared with the other MS patients (data not shown). Furthermore, excluding these patients from the statistical analyses provided in Fig. 2 revealed only minor changes in the median values of the respective groups and had no influence on statistical significance levels (data not shown). These data indicate that concomitant local pathology as well as systemic inflammation may result in increased oxidative stress and Nrf2 expression within the brain white matter, but that these factors had no major influence on the Nrf2 expression patterns related to MS pathology.

## Discussion

The concept of neuroprotective treatment of multiple sclerosis patients with fumarates is based on their anti-inflammatory actions [38], mediated for instance by inhibition of microglia activation [35] and on their induction of the transcription factor Nrf2 and of endogenous downstream anti-oxidant defense mechanisms [28, 37]. This treatment paradigm is supported by the observation that oxidative injury is a major factor involved in demyelination and neurodegeneration in the CNS of MS patients.

The potential anti-oxidative protective effect of fumarates may depend on the pre-existent expression levels of Nrf2 in cells or tissues. Fumarates induce Nrf2 and are neuroprotective in conditions of no or low basic expression of Nrf2, such as during EAE [28]. However, in conditions of pre-existing profound oxidative stress, high basic expression of Nrf2 can be expected, which may not be further increased by fumarate treatment. Additional fumarate-induced cells stress in oligodendrocytes or neurons already exposed to severe oxidative injury might even increase demyelination or neurodegeneration. Further induction of Nrf2 in cells, which already express this protein at high levels, may propagate cell death by interacting with another promotor element through the induction of KLF-9 [46]. Thus, anti-oxidant effects of fumarates may differ profoundly depending on the degree of oxidative injury and pre-existing expression levels of Nrf2 in the tissue.

For these reasons, it is important to know in detail the extent and patterns of Nrf2 expression in MS patients at different disease stages and in different lesion types. So far, only two studies have addressed this question and concluded that its low expression may be insufficient to protect the tissue against oxidative injury. In our present study, we found very prominent Nrf2 expression predominantly in oligodendrocytes at sites of initial demyelination in active MS lesions. Within these cells, Nrf2 appeared to be functionally active, as indicated by the expression of HO-1 in the same cells and the selective up-regulation of other Nrf2-responsive genes in microdissected lesion areas with initial stages of demyelination and tissue injury. As in our study, presence of HO-1 in oligodendrocytes has been previously described in active MS lesions [40]. Excessive Nrf2 expression was seen in cells with signs of cell degeneration, such as the loss of cytoplasmic marker proteins and nuclear DNA fragmentation. Furthermore, the liberation of AIF from mitochondrial stores into the cytoplasm and nucleus, as seen in cells with excessive Nrf2 expression, has been already described as a major pathway for oxidative cell death in MS lesions [15, 44]. High Nrf2 expression was present in active lesions of acute and relapsing MS as well as in slowly expanding lesions in progressive MS. Nrf2-positive astrocytes and macrophages were seen mainly in later stages of active lesions characterized by complete demyelination and profound oligodendrocyte loss. Such a pattern of Nrf2 immunoreactivity has been reported previously by van Horssen et al. [41]. In contrast to this report, we also included in our study patients with acute MS and highly active lesions and we also used very stringent criteria for identification of lesional activity in patients with progressive MS based on the presence of early myelin degradation products in macrophages. Nrf2 expression in MS is fundamentally different from that seen in EAE, where it is sparse or absent even in highly active lesions, but is induced in oligodendrocytes, astrocytes, macrophages and neurons after fumarate treatment [28].

So far, we convincingly found Nrf2 in mature oligodendrocytes in active MS lesions, but we cannot draw conclusions on its expression in oligodendrocyte progenitor cells (OPCs). Although a variety of markers for OPCs are currently available, none of them work with convincing reliability in archival autopsy tissue with variable post mortem autolysis time and formaldehyde fixation time.

Expression of Nrf2 in neurons has been described in control patients and in various neurodegenerative diseases, but it was concluded that its expression may be insufficient to prevent neuronal degeneration [36]. In our study, the nuclear Nrf2 reactivity in neurons in MS lesions was low or absent, even in gray matter lesions with extensive acute oxidative injury. Only exceptionally, neurons with morphological features of acute (terminal) hypoxic injury revealed prominent Nrf2 up-regulation and nuclear translocation. Interestingly, within the same sections and lesion areas, oligodendrocytes with abundant Nrf2 expression were located in close vicinity to Nrf2-negative neurons. Thus, the lack of Nrf2 expression in neurons is not due to different lesion environments between gray and white matter, but seems to reflect an intrinsic difference in the reaction to inflammation, demyelinating and oxidative stress between neurons and oligodendrocytes. The low induction of Nrf2 in cortical neurons was also reflected by the lack of induction of most Nrf2-responsive anti-oxidant molecules and may also be related to the down-regulated expression of another transcription factor (peroxisome proliferator-activated receptor gamma, coactivator 1 alpha, PGC-1α, encoded by the *PPARGC1A* gene), which regulates mitochondrial anti-oxidant mechanisms [45]. In addition, it has been recently shown that Nrf2 expression is repressed in neurons in the adult nervous system by epigenetic inactivation of its promotor [3]. Profound induction of Nrf2 has been seen in neurons in vitro [37] and in vivo in EAE animals treated with fumarates [28] and protected neurons against oxidative injury [37]. However, as in MS patients, in PML patients treated with fumarate we did not observe an increase of nuclear Nrf2 reactivity in cortical neurons, despite increased nuclear immunoreactivity within the white matter lesions of the same patients [31]. This may be of major importance for the interpretation of treatment effects in patients with progressive MS, since gray matter damage appears to play a key role in disability progression [7, 13].

A limitation of our study is that pathology does not provide direct information on the temporal pattern of Nrf2 expression. Thus, induction of Nrf2 expression by fumarates in oligodendrocytes before new lesions are formed may be protective, while its further stimulation in active lesions might have no or even opposite effects. In addition, up-regulation of anti-oxidant defense molecules in astrocyte may indirectly exert neuroprotective effects in adjacent cells such as neurons [33]. This view is further supported by previous studies, which showed that anti-oxidant molecules such as NAD(P)H:quinone oxidoreductase 1, thioredoxin-2, or thioredoxin reductase are mainly expressed in astrocytes, while these anti-oxidant defense mechanisms are less effective in neurons [6, 12, 33, 42]. Overall, our study provides further evidence for an important role of oxidative stress as a mechanism of demyelination and neurodegeneration in MS and for extensive up-regulation of anti-oxidant defense molecules at least in active white matter lesions.

## Electronic supplementary material

Supplementary material 1 (PDF 305 kb)

## Abbreviations

MS: Multiple sclerosis
RRMS: Relapsing–remitting MS
PPMS: Primary progressive MS
SPMS: Secondary progressive MS
NWM: Normal white matter
NAWM: Normal-appearing white matter
PPWM: Periplaque white matter
Nrf2: Nuclear factor (erythroid-derived 2)-like 2
HO-1: Heme oxygenase 1
AIF: Apoptosis-inducing factor
CAII: Carbonic anhydrase II
GFAP: Glial fibrillary acidic protein
KLF-9: Kruppel-like factor 9
