# Supplementary material for: Cell type-specific Nrf2 expression in multiple sclerosis lesions
Source: Acta Neuropathol. 2015 Jun 19;130(2):263–77. doi: 10.1007/s00401-015-1452-x (PMC4503875; doi:10.1007/s00401-015-1452-x)
Supplement: Supplementary file 1 — Supplementary material 1 (PDF 305 kb) [file 401_2015_1452_MOESM1_ESM.pdf]

Cell type-specific Nrf2 expression in MS lesions

Acta Neuropathologica

Simon Licht-Mayer[1], Isabella Wimmer[1], Sarah Traffehn[2], Imke Metz[2], Wolfgang Brück[2], Jan Bauer[1], Monika Bradl[1], Hans Lassmann[1]

1: Department of Neuroimmunology, Center for Brain Research, Medical University of Vienna, Austria

2: Institute of Neuropathology, University of Göttingen, Germany

All correspondence to:
Prof. Dr. Hans Lassmann
Center for Brain Research
Medical University of Vienna
Spitalgasse 4
A-1090 Wien
Tel.: +43 1 40160 34200
Fax.: +43 1 40160 934203
e-mail: hans.lassmann@meduniwien.ac.at

Supplementary Figure 1:

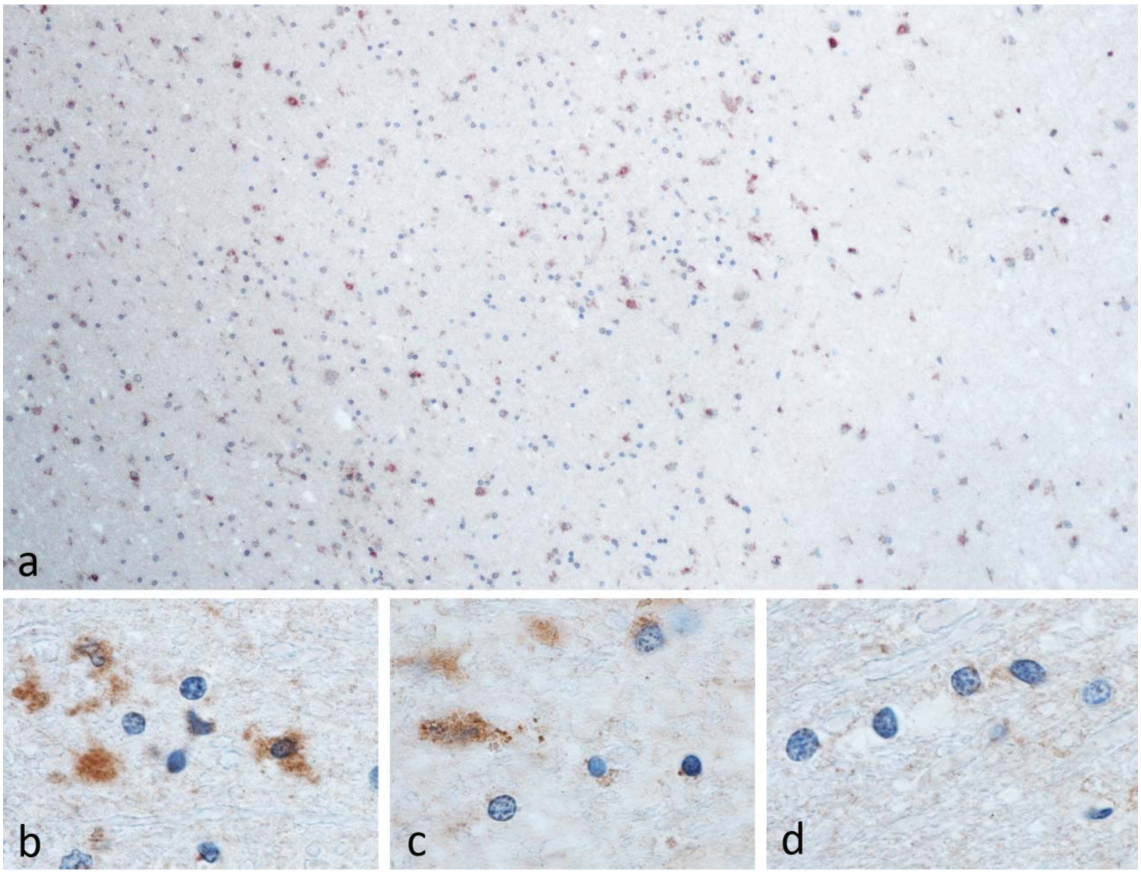

Slowly expanding (smoldering) lesion in primary progressive multiple sclerosis (MS21) stained for KEAP1 (immunohistochemistry). a) KEAP1 reactivity is seen in cells at the active lesion edge (left), while only few cells are present in the inactive lesion center (x 100); b-d) Higher magnification images from the same tissue sections showing immunoreactivity exclusively in the cytoplasm, but no reactivity within the nucleus. Reactivity is seen in different cells, including astrocytes (b) and oligodendrocytes and macrophages (c). In the normal appearing white matter, distant from active lesions immunoreactivity within cells is very low (d); x: 1000.

Supplementary Table 1: Expression of Nrf2-responsive genes in multiple sclerosis

	MS white matter				MS cortex			
	Expression	Fold-change			Fold-change			
	Control	PPWM/C	Initial/C	Active/C	MS/C	MS38/C		
Group 1								
PGD [4]	11.1	-1.10	20.62	-1.35	-2.00	-1.38	NM_002631	Phosphogluconate dehydrogenase
GNAI2 [9]	6.0	2.27	12.60	3.25	-1.32	-1.67	NM_002070	Nucleotide binding protein (G protein), alpha inhibiting activity polypeptide 2, transcript variant 1
FTH1 [1, 2, 4, 9]	8.8	1.45	6.04	1.41	1.40	2.23	NM_002032	Ferritin, heavy polypeptide 1
ERP29 [2]	6.4	2.87	4.41	4.76	-1.99	-2.31	NM_006817	Endoplasmic reticulum protein 29, transcript variant 1
TXN [1, 2, 7, 9]	7.2	1.60	4.02	-1.23	7.09	17.71	NM_003329	Thioredoxin, transcript variant 1
ANKRD11 [1]	5.9	2.21	3.91	1.01	-1.57	-1.08	NM_013275	Ankyrin repeat domain 11
GATA3 [1]	12.5	1.25	3.41	1.93	-3.71	-4.21	NM_001002295	GATA binding protein 3, transcript variant 1
FTL [1, 2, 4, 9]	4.2	5.02	3.34	3.47	-1.07	1.29	NM_000146	Ferritin, light polypeptide
HBG1 [1]	5.4	1.26	3.10	2.48	-1.04	-1.67	NM_000559	Hemoglobin, gamma A
BACH1 [6]	4.1	-1.11	2.64	-1.02	-1.66	-2.25	NM_206866	BTB and CNC homology 1, basic leucine zipper transcription factor 1, transcript variant 1
PPARG [1]	7.9	-1.14	2.29	-1.04	-1.18	-1.40	NM_138711	Peroxisome proliferator-activated receptor gamma, transcript variant 3
GCS [5]	5.0	1.01	2.10	1.75	-1.05	-1.15	M90656	Gamma-glutamylcysteine synthetase
PRDX1 [1, 2, 4, 7]	10.4	-1.12	2.06	1.43	-2.52	-3.03	NM_002574	Peroxiredoxin 1, transcript variant 1
UNKL [1]	10.0	4.00	1.96	1.42	-1.52	-1.35	NM_001037125	Unkempt homolog (Drosophila)-like, transcript variant 2
USP14 [2, 7]	4.1	1.38	1.93	1.20	-1.68	-1.43	NM_005151	Ubiquitin specific peptidase 14 (tRNA-guanine transglycosylase), transcript variant 1
TALDO1 [1]	7.5	-1.68	1.72	-1.00	2.75	3.07	NM_006755	Transaldolase 1
FECH [1]	6.3	1.02	1.71	1.47	1.78	1.70	NM_001012515	Ferrochelatase (FECH), transcript variant 1
AMBP [1]	5.0	4.59	1.37	1.55	-2.82	-2.86	NM_001633	Alpha-1-microglobulin/bikunin precursor
Group 2								
TXNRD2 [3]	18.9	1.88	5.85	1.17	-1.53	-1.96	NM_006440	Thioredoxin reductase 2
CCT7 [2]	18.1	3.70	5.45	14.30	-5.54	-2.98	NM_006429	Chaperonin containing TCP1, subunit 7 (eta), transcript variant 1
OSGIN1 [1]	14.8	-1.42	5.32	-1.19	-1.04	-1.15	NM_182981	Oxidative stress induced growth inhibitor 1
GNPDA1 [1]	28.1	6.61	5.10	3.83	-3.69	-4.46	NM_005471	Glucosamine-6-phosphate deaminase 1
HMOX1 [1, 2, 4, 7, 9]	1689.4	-2.44	3.85	2.17	129.23	254.91	NM_002133	Heme oxygenase (decycling) 1
BMP10 [1]	22.6	2.64	2.70	-1.15	-1.09	-1.70	NM_014482	Bone morphogenetic protein 10
GSTP1 [1, 4, 9]	22441.9	-4.92	2.40	1.78	4.09	4.54	NM_000852	Glutathione S-transferase pi 1
SLC3A2 [1]	124.2	-12.69	2.34	2.77	2.96	3.54	NM_001012662	Solute carrier family 3 (activators of dibasic and neutral amino acid transport), member 2, transcript variant 2
MT2A [1]	201.9	-9.27	2.16	1.83	7.82	16.38	NM_005953	Metallothionein 2A
ALDH2 [10]	14.6	-1.86	2.05	1.53	1.11	1.39	NM_000690	Aldehyde dehydrogenase 2 family (mitochondrial), transcript variant 1
CYP1A1 [2]	28.7	1.17	1.76	1.64	-1.61	-1.51	NM_000499	Cytochrome P450, family 1, subfamily A, polypeptide 1
SOD3 [2]	71.0	-1.96	1.63	-1.01	3.08	8.01	NM_003102	Superoxide dismutase 3, extracellular

KIFC3 [1]	167.0	1.69	1.57	1.70	-1.87	-1.44	NM_005550	Kinesin family member C3, transcript variant 1
VCP [2]	23.1	1.05	1.43	1.08	2.07	3.41	NM_007126	Valosin containing protein
MAFG [1, 7, 9]	181.2	1.73	1.28	1.01	-1.24	-1.13	NM_002359	V-maf musculoaponeurotic fibrosarcoma oncogene homolog G (avian), transcript variant 1
ME1 [1, 4, 10]	90,7	-2,12	1,26	1,32	-2,01	-4,99	NM_002395	Malic enzyme 1, NADP(+)-dependent, cytosolic
HERPUD1 [1]	121.3	-22.51	1.13	-1.21	3.54	5.36	NM_014685	Homocysteine-inducible, endoplasmic reticulum stress-inducible, ubiquitin-like domain member 1, transcript variant 1
IDH1 [4]	14.3	2.47	1.01	1.14	-1.79	-2.02	NM_005896	Isocitrate dehydrogenase 1 (NADP+), soluble
MT1E [2]	208.0	-3.20	-1.07	1.42	2.36	2.57	NM_175617	Metallothionein 1E
CLPP [2]	86.1	-5.26	-1.15	-1.62	7.37	9.50	NM_006012	ClpP caseinolytic peptidase, ATP-dependent, proteolytic subunit homolog (E. coli)
SOD1 [3]	102.9	-3.47	-1.24	16.87	-2.07	-3.05	NM_000454	Superoxide dismutase 1, soluble
GPX2 [2, 4, 9]	851.1	1.20	-1.26	1.02	-3.02	-3.63	NM_002083	Glutathione peroxidase 2 (gastrointestinal)
STIP1 [2]	13.2	-1.42	-1.29	2.05	-1.41	-1.71	NM_006819	Stress-induced-phosphoprotein 1 (STIP1)
UNKL [1]	157.5	-1.43	-1.35	1.14	1.09	1.78	NM_001193388	Unkempt homolog (Drosophila)-like, transcript variant 1
GSR [1, 2]	10.2	1.02	-1.41	-1.64	-1.56	-1.09	NM_000637	Glutathione reductase, transcript variant 1
UBB [2]	184.2	-1.12	-1.45	-1.02	-1.16	-1.29	NM_018955	Ubiquitin B
SQSTM1 [1, 2, 7]	8.8	4.41	-1.56	1.44	3.56	1.76	NM_003900	Sequestosome 1, transcript variant 1
RXRA [1]	61.4	-1.97	-1.59	-1.53	-2.05	-1.90	NM_002957	Retinoid X receptor, alpha
GSTA3 [4, 9]	75.2	-2.11	-1.64	-1.24	1.30	-1.09	NM_000847	Glutathione S-transferase alpha 3
GPI [1]	529.6	-12.10	-1.83	-3.56	1.41	1.86	NM_000175	Glucose-6-phosphate isomerase, transcript variant 2
FMO1 [2]	403.4	-1.40	-1.85	-1.19	-1.04	-1.51	NM_002021	Flavin containing monooxygenase 1
MT1A [2]	167.0	-1.28	-1.97	-1.77	1.93	1.31	NM_005946	Metallothionein 1A
ETS1 [8, 9]	10.3	-1.48	-2.00	-1.60	-1.37	-1.87	NM_005238	V-ets erythroblastosis virus E26 oncogene homolog 1 (avian), transcript variant 2
ABCB6 [1, 2]	25.0	-2.15	-2.07	-3.31	1.59	1.95	NM_005689	ATP-binding cassette, sub-family B (MDR/TAP), member 6
HBE1 [1]	46.5	-1.48	-2.08	-1.48	1.34	-1.19	NM_005330	Hemoglobin, epsilon 1
FKBP5 [2]	21.5	-1.33	-3.04	-1.06	-1.03	1.60	NM_004117	FK506 binding protein 5, transcript variant 1
TFE3 [1]	908.7	1.20	-7.41	-2.53	-7.44	-15.56	NM_006521	Transcription factor binding to IGHM enhancer 3
Group 3								
SH3TC1 [1]	4.0	1.02	1.48	1.17	-1.05	-1.03	NM_018986	SH3 domain and tetratricopeptide repeats 1
CBR1 [2]	4.6	2.05	1.45	1.22	-3.77	-3.35	NM_001757	Carbonyl reductase 1
NUP62 [1]	3.8	2.34	1.39	1.01	3.90	6.82	NM_153719	Nucleoporin 62kDa, transcript variant 1
CAT [2]	5.0	1.60	1.37	1.89	-1.37	-2.61	NM_001752	Catalase
NDUFAF4 [1]	4.2	-1.03	1.37	1.02	n.d.	n.d.	NM_014165	NADH dehydrogenase (ubiquinone) 1 alpha subcomplex, assembly factor 4
CPEB3 [1]	3.9	1.14	1.37	2.33	1.35	-1.32	NM_014912	Cytoplasmic polyadenylation element binding protein 3, transcript variant 1
DNAJA1 [2, 10]	12.9	1.11	1.31	-1.09	-1.38	-1.91	NM_001539	DnaJ (Hsp40) homolog, subfamily A, member 1
HIST1H4H [1]	4.0	1.10	1.30	1.06	1.50	-1.22	NM_003543	Histone cluster 1, H4h
NQO2 [5, 9]	3.8	-1.05	1.29	1.09	1.19	2.31	NM_000904	NAD(P)H dehydrogenase, quinone 2
PPARGC1B [1]	4.1	1.05	1.29	1.05	1.42	1.43	NM_133263	Peroxisome proliferator-activated receptor gamma, coactivator 1 beta, transcript variant 1
RBX1 [5]	5.2	2.04	1.23	1.13	-1.53	-1.26	NM_014248	Ring-box 1, E3 ubiquitin protein ligase
GCLC [1, 2, 4, 9]	3.8	1.02	1.20	1.09	6.80	6.03	NM_001498	Glutamate-cysteine ligase, catalytic subunit, transcript variant 1

GSTA5 [4]	3.7	1.21	1.19	1.03	-3.79	-4.13	NM_153699	Glutathione S-transferase alpha 5
CPEB2 [1]	5.2	-1.07	1.17	-1.11	1.17	-1.41	NM_182485	Cytoplasmic polyadenylation element binding protein 2, transcript variant B
TKT [1]	4.1	-1.01	1.16	-1.03	9.48	13.50	NM_001064	Transketolase, transcript variant 1
G6PD [4]	5.7	-1.36	1.12	-1.35	1.63	-1.57	NM_000402	Glucose-6-phosphate dehydrogenase, transcript variant 1
AIFM2 [1]	8.2	-1.18	1.10	-1.13	1.79	1.55	NM_032797	Apoptosis-inducing factor, mitochondrion-associated, transcript variant 2
UNKL [1]	4.1	1.08	1.08	1.23	n.d.	n.d.	NM_023076	Unkempt homolog (Drosophila)-like, transcript variant 3
GSTA1 [4, 9]	3.8	1.18	1.07	1.07	1.44	-1.33	NM_145740	Glutathione S-transferase alpha
PIR [1]	3.6	-1.01	1.06	1.40	515.60	2.92	NM_003662	Pirin (iron-binding nuclear protein), transcript variant 1
GPD2 [1]	4.2	1.20	1.05	1.08	1.39	1.46	NM_000408	Glycerol-3-phosphate dehydrogenase 2 (mitochondrial), transcript variant 2
PTPLAD1 [1]	3.7	1.12	1.04	1.05	1.57	3.23	NM_016395	Protein tyrosine phosphatase-like A domain containing 1
PPIB [2]	3.7	1.25	1.04	1.19	-1.21	-1.22	NM_000942	Peptidylprolyl isomerase B (cyclophilin B)
GCLM [2, 4, 7]	5.9	-1.12	1.03	-1.26	1.06	-1.33	NM_002061	Glutamate-cysteine ligase, modifier subunit
NQO1 [1, 2, 3, 4, 5, 7]	3.7	1.11	1.01	1.10	-2.51	-1.95	NM_000903	NAD(P)H dehydrogenase, quinone 1, transcript variant 1
TXNRD1 [1, 4, 7, 9]	3.9	-1.02	1.01	-1.01	1.70	2.32	NM_003330	Thioredoxin reductase 1, transcript variant 1
HTATIP2 [1]	3.9	-1.05	1.01	-1.01	-1.31	-2.61	NM_001098523	HIV-1 Tat interactive protein 2, 30kDa, transcript variant 5
CUL3 [5]	4.2	1.20	-1.00	1.08	1.53	2.27	NM_003590	Cullin 3
BACH1 [6]	4.8	1.07	-1.04	-1.08	-3.11	-3.42	NR_027655	BTB and CNC homology 1, basic leucine zipper transcription factor 1, transcript variant 3
tAKR [2]	4.5	1.07	-1.05	1.03	-2.16	-2.10	NR_026743	Aldo-keto reductase, truncated
KEAP1 [1, 5, 7]	4.5	-1.04	-1.08	-1.10	3.31	2.37	NM_203500	Kelch-like ECH-associated protein 1, transcript variant 1
PSMA3 [1]	4.1	-1.13	-1.12	-1.18	2.27	3.17	NM_148976	Proteasome (prosome, macropain) subunit, alpha type, 1, transcript variant 1
GSTA2 [4]	5.5	-1.23	-1.34	-1.28	-2.19	-2.67	NM_000846	Glutathione S-transferase alpha 2
GSR [1, 4, 7]	10.2	1.02	-1.41	-1.64	-1.56	-1.09	NM_000637	Glutathione reductase, transcript variant 1
AOX1 [2]	5.9	1.11	-1.41	1.16	1.24	1.49	NM_001159	Aldehyde oxidase 1
PPARGC1A [11]	5.6	1.01	-1.46	-1.26	-1.80	-1.80	NM_013261	Peroxisome proliferator-activated receptor gamma, coactivator 1 alpha
HTATIP2 [1]	6.6	1.04	-1.63	-1.31	-1.15	-1.07	NM_006410	HIV-1 Tat interactive protein 2, 30kDa, transcript variant 2
EPHX1 [1, 7]	7.0	-1.57	-1.82	-1.59	-2.29	-1.50	NM_000120	Epoxide hydrolase 1, microsomal (xenobiotic), transcript variant 1

The first column of the table gives the official gene symbols. Additionally, the respective literature is referenced in superscript numbers. In the second column, the mean basal expression levels within the normal white matter of control cases is depicted. The following three columns show the calculated gene expression fold-changes within the periplaque white matter (PP), initial (prephagocytic) lesions (I)and active demyelinating lesions (A) in comparison with the normal white matter of controls. Thereafter, the calculated gene expression fold-changes within active cortical lesions from all three evaluated MS cases (All) or a single MS case (MS15) in comparison with the normal grey matter of control cases are shown.

The Nrf2-responsive genes are categorized according to their basal expression in the NWM of controls and expression fold-changes in MS lesions in comparison with the respective control tissue. **Group 1** represents Nrf2-responsive genes that show a low basal expression in the NWM of controls, but were up-regulated in the zones of initial demyelination, while their expression was lower in the NAWM and the demyelinated part of the active lesion. The second pattern (**Group**

2) consists of genes, which are expressed in high levels in the NWM of controls. Genes within **group 3** show a very low basal expression in the NWM of controls and do not show any dynamic expression changes in MS lesions.

Supplementary References:

1 Chorley BN, Campbell MR, Wang X, Karaca M, Sambandan D, Bangura F, Xue P, Pi J, Kleeberger SR, Bell DA. Identification of novel NRF2-regulated genes by ChIP-Seq: influence on retinoid X receptor alpha. Nucleic Acids Res. 2012 Aug;40(15):7416-29. doi: 10.1093/nar/gks409. Epub 2012 May 11.

2 Córdova EJ, Martínez-Hernández A, Uribe-Figueroa L, Centeno F, Morales-Marín M[2], Koneru H, Coleman MA, Orozco L. The NRF2-KEAP1 pathway is an early responsive gene network in arsenic exposed lymphoblastoid cells. PLoS One. 2014 Feb 7;9(2):e88069. doi: 10.1371/journal.pone.0088069. eCollection 2014.

3 Taylor RC, Acquaah-Mensah G, Singhal M, Malhotra D, Biswal S. Network inference algorithms elucidate Nrf2 regulation of mouse lung oxidative stress. PLoS Comput Biol. 2008 Aug 29;4(8):e1000166. doi: 10.1371/journal.pcbi.1000166.

4 Gorrini C, Harris IS, Mak TW. Modulation of oxidative stress as an anticancer strategy. Nat Rev Drug Discov. 2013 Dec;12(12):931-47. doi: 10.1038/nrd4002.

5 Niture SK, Khatri R, Jaiswal AK. Regulation of Nrf2-an update. Free Radic Biol Med. 2014 Jan;66:36-44. doi: 10.1016/j.freeradbiomed.2013.02.008. Epub 2013 Feb 19.

6 Jyrkkänen HK, Kuosmanen S, Heinäniemi M, Laitinen H, Kansanen E, Mella-Aho E, Leinonen H, Ylä-Herttuala S, Levonen AL. Novel insights into the regulation of antioxidant-response-element-mediated gene expression by electrophiles: induction of the transcriptional repressor BACH1 by Nrf2. Biochem J. 2011 Dec 1;440(2):167-74. doi: 10.1042/BJ20110526.

7 Cook AL, Vitale AM, Ravishankar S, Matigian N, Sutherland GT, Shan J, Sutharsan R, Perry C, Silburn PA, Mellick GD, Whitelaw ML, Wells CA, Mackay-Sim A, Wood SA. NRF2 activation restores disease related metabolic deficiencies in olfactory neurosphere-derived cells from patients with sporadic Parkinson's disease. PLoS One. 2011;6(7):e21907. doi: 10.1371/journal.pone.0021907. Epub 2011 Jul 1.

8 Wilson LA, Gemin A, Espiritu R, Singh G. ets-1 is transcriptionally up-regulated by H2O2 via an antioxidant response element. FASEB J. 2005 Dec;19(14):2085-7. Epub 2005 Oct 18.

9 Malhotra D, Portales-Casamar E, Singh A, Srivastava S, Arenillas D, Happel C, Shyr C, Wakabayashi N, Kensler TW, Wasserman WW, Biswal S. Global mapping of binding sites for Nrf2 identifies novel targets in cell survival response through ChIP-Seq profiling and network analysis. Nucleic Acids Res. 2010 Sep;38(17):5718-34. doi: 10.1093/nar/gkq212. Epub 2010 May 11.

10 Thimmulappa RK, Mai KH, Srisuma S, Kensler TW, Yamamoto M, Biswal S. Identification of Nrf2-regulated genes induced by the chemopreventive agent sulforaphane by oligonucleotide microarray. Cancer Res. 2002 Sep 15;62(18):5196-203.

11 Whitman SA, Long M, Wondrak GT, Zheng H, Zhang DD. Nrf2 modulates contractile and metabolic properties of skeletal muscle in streptozotocin-induced diabetic atrophy. Exp Cell Res. 2013 Oct 15;319(17):2673-83. doi: 10.1016/j.yexcr.2013.07.015. Epub 2013 Jul 27.
